# Influenza Vaccination of Patients With Systemic Lupus Erythematosus (SLE) and Rheumatoid Arthritis (RA)

**DOI:** 10.1080/17402520600800820

**Published:** 2006

**Authors:** Ljudmila Stojanovich

**Affiliations:** “Bezhanijska kosa” University Medical Center Belgrade University Serbia

## Abstract

The role of influenza vaccination in patients suffering from autoimmune diseases, including systemic lupus erythematosus (SLE) and rheumatoid arthritis (RA), has long been a subject of discussion. The risk of exacerbation of the main disease following vaccination is of particular concern, and needs to be carefully evaluated against the risk of disease flares as a result of infections. Our study included 69 SLE patients and 54 RA patients, all in stable condition. We split the groups into two subgroups each: patients in SLE_1_ (23 patients) and RA_1_ (23 patients) received the flu vaccine (“Vaxigrip”, Aventis Pasteur) in November 2003. Patients in SLE_2_ (46 patients) and RA_2_ (31 patients) were not vaccinated. Throughout the following year, we studied parameters of disease activity and the occurrence of viral respiratory and bacterial infections in our patients. The vaccine was well tolerated in all cases. Vaccinated patients had significantly fewer occurrences of infections. Every viral and bacterial infection resulted in the worsening of the main disease. We believe that influenza vaccine is indicated for SLE and RA patients in stable condition. However, this decision must be made on a patient-by-patient basis. We plan to continue our study with the goal of formulating a better protocole for the clinical practice.


*Clinical & Developmental Immunology*, June–December 2006; 13(2–4): 373–375

Taylor & Francis

Taylor & Francis Group

### Influenza vaccination of patients with systemic lupus erythematosus (SLE) and rheumatoid arthritis (RA)

LJUDMILA STOJANOVICH

*"Bezhanijska kosa" University Medical Center, Belgrade University, Serbia*

**Abstract**
The role of influenza vaccination in patients suffering from autoimmune diseases, including systemic lupus erythematosus (SLE) and rheumatoid arthritis (RA), has long been a subject of discussion. The risk of exacerbation of the main disease following vaccination is of particular concern, and needs to be carefully evaluated against the risk of disease flares as a result of infections. Our study included 69 SLE patients and 54 RA patients, all in stable condition. We split the groups into two subgroups each: patients in $SLE_1$ (23 patients) and $RA_1$ (23 patients) received the flu vaccine ("Vaxigrip", Aventis Pasteur) in November 2003. Patients in $SLE_2$ (46 patients) and $RA_2$ (31 patients) were not vaccinated. Throughout the following year, we studied parameters of disease activity and the occurrence of viral respiratory and bacterial infections in our patients. The vaccine was well tolerated in all cases. Vaccinated patients had significantly fewer occurrences of infections. Every viral and bacterial infection resulted in the worsening of the main disease. We believe that influenza vaccine is indicated for SLE and RA patients in stable condition. However, this decision must be made on a patient-by-patient basis. We plan to continue our study with the goal of formulating a better protocole for the clinical practice.

**Keywords:** *Influenza vaccination, rheumatoid arthritis, systemic lupus erythematosus, autoimmune diseases*

### Introduction

Vaccination against influenza is the primary strategy to reduce the mortality and morbidity associated with influenza. The vaccine is primarily recommended for persons at increased risk of severe influenza such as immunocompromised subjects, including patients with autoimmune diseases (Herron et al. 1979; Shoenfeld and Rose 2004).

The role of influenza vaccination in patients suffering from autoimmune diseases, including systemic lupus erythematosus (SLE) and rheumatoid arthritis (RA), has long been a subject of discussion (Nosal 2000). Etiology of autoimmune diseases is still unknown, and among others, two possible triggers are cited in the literature: infections and vaccination (Rose 1998; Sibilia and Maillefert 2002; Aron-Maor and Shoenfeld 2004). An infection can induce or trigger autoimmune disease (Doran et al. 2002; Glück et al. 2005). Viral infections have specifically been causally associated with SLE (Zandman-Goddard and Shoenfeld 2004), and there are documented cases of SLE presenting itself after vaccination (Shoenfeld and Rose 2004). However, it has also been documented that the swine-flu influenza vaccine was well tolerated in SLE and RA patients (Avery 1999; Stojanovich 2005; Del Porto et al. 2006), albeit with a lower response to immunization compared to healthy controls.

However, same case reports suggest an association between influenza vaccination and the development or exacerbation of chronic autoimmune disorders such as RA, SLE (Blumberg et al. 1980) and vasculitis (Iyngkaran et al. 2003).

Patients with autoimmune rheumatic diseases run twice as high a risk of infection compared to healthy controls (Doran et al. 2002). This is due in part to the immunoregulatory abnormalities associated with the disease itself, but also in large part to the immunosuppressive therapy administered to these patients in the interest of delaying joint destruction. The effect of disease-modifying therapies on the

Correspondence: L. Stojanovich, "Bezhanijska Kosa" University Medical Center, Belgrade University, Bezanijski put BB, Novi Beograd, Belgrade 11080, Serbia. Tel: 381 11 3010 777. Fax: 381 11 606 520. E-mail: ljudmila.stojanovich@rvkds.net

ISSN 1740-2522 print/ISSN 1740-2530 online © 2006 Taylor & Francis
DOI: 10.1080/17402520600800820

immune system may be quite dramatic, especially if some of the more aggressive protocols are administered that may lead to opportunistic infections otherwise only seen in patients with advanced HIV infection (Glück et al. 2005).

### Materials and methods

Our study included 69 patients with SLE (mean age 44.74 ± 13.58) and 54 patients with RA (mean age 57.78 ± 12.42). We further split both groups into two subgroups each: subgroups $SLE_1$ (23 patients, 33.33%) and $RA_1$ (23 patients, 42.59%) included patients with SLE and RA, respectively, who received the flu vaccine ("Vaxigrip", Aventis Pasteur). Subgroups $SLE_2$ (46 patients, 66.67%) and $RA_2$ (31 patients, 57.41%) were comprised of patients who were not vaccinated against flu. The two pairs of subgroups were comparable with respect to age, gender, disease activity, manifestations of the main disease, and other parameters (such as immunoserological parameters, etc). Patients from groups $SLE_1$ and $RA_1$ were all vaccinated in November 2003. Throughout the following year, we studied parameters of disease activity (SLEDAI for SLE) and the occurrence of viral respiratory and bacterial infections in our patients.

### Results

The vaccine was well tolerated by all patients in groups $SLE_1$ and $RA_1$. The following occurrences of viral respiratory and bacterial infections were registered in our patients: 1 (2.17%) patient from $SLE_2$ had pneumonia; 2 (8.7%) patients from $SLE_1$ and 17 (36.97%) patients from $SLE_2$ suffered from acute bronchitis that required antibiotic treatment. Various viral respiratory infections occurred significantly more often in $SLE_2$ compared to $SLE_1$, with $p < 0.005$. However, these infections also correlated with age (patients older than 30 years of age) with $p = 0.04783$. The occurrence of bacterial and viral respiratory infections in our SLE population is summarized in Table I. In no single case did we witness worsening of main disease after vaccination. Unfortunately, this was not the case in $SLE_2$, where primary symptoms of SLE worsened after treatments of viral and bacterial infections with statistical significance ($p = 0.008$). A similar tendency was registered in patients with RA. No pneumonia occurred in either $RA_1$ or $RA_2$.

Table I. Systemic lupus erythematosus (SLE).

| Disease | $SLE_1$ $N$ (%) | $SLE_2$ $N$ (%) | $p$ |
|---|---|---|---|
| Pneumonia | 0(0) | 1(8.7) | <0.05 |
| Acute bronchitis | 1(4.3) | 17(36.9) | <0.05 |
| Viral respiratory infections | 5(21.7) | 21(45.6) | <0.05 |

Table II. Rheumatoid arthritis (RA).

| Disease | $RA_1$ $N$ (%) | $RA_2$ $N$ (%) | $p$ |
|---|---|---|---|
| Pneumonia | 0(0) | 0(0) | 0 |
| Acute bronchitis | 1(4.3) | 7(22.6) | <0.05 |
| Viral respiratory infections | 2(8.7) | 19(61.3) | <0.05 |

However, acute bronchitis that required antibiotic treatment was diagnosed in only 1 (4.35%) patient in $RA_1$, compared to 7 (22.58%) patients in $RA_2$. Various viral respiratory infections were seen in 2 (8.70%) patients in $RA_1$, compared to 19 (61.29%) patients in $RA_2$, a statistically significant difference (Table II). Every viral and bacterial infection in our autoimmune patients diseases resulted in the worsening of the main disease, and required a correction of immune-suppressants.

### Discussion

It is important to note that infections are of particular concern to autoimmune patients (Shoenfeld and Rose 2004). Not only is the course of the main disease often aversely affected by the infection, but also cytostatic therapy has to be paused during that time, which may itself lead to exacerbation of the main disease (Fomin et al. 2006). To make matters worse, autoimmune patients receive long-term treatment with steroids and immunosuppressant, which makes them more susceptible to infections such as influenza.

The goal of our study is two-fold. On the one hand, we study the risks associated with vaccination by a modern influenza vaccine in SLE and RA patients in stable condition. Additionally, we analyse the dangers posed by viral respiratory and bacterial infections to these patients.

We have shown that vaccination against influenza was safe and generated a good humoral response in SLE and RA.

Although yearly influenza vaccination is now recommended for patients with RA, a paucity of long-term studies deal with the influence of repeated vaccination upon both the incidence and exacerbation of pre-existing systemic rheumatic diseases (Kurland et al. 1984; Gavaghan and Webber 1993). If a true increase in relative risk exists, albeit small, then an increased frequency of such complications might be anticipated. Although for the vast majority of patients the benefits of regular influenza vaccination will likely far exceed its potential risks, we still advise appropriate monitoring of individuals with known systemic rheumatic diseases, early reporting of suspected adverse events associated with vaccination, and consideration of large prospective studies to determine the actual risks of currently recommended vaccination protocols (Chalmers et al. 1994; Cimmino et al. 1995; Avery 1999).

Despite this recommendation, the uptake of vaccination in autoimmune patients is still sub-optimal, mostly because the patients have not been offered the vaccine. Reduced compliance with immunization against influenza is related to concerns about the vaccine's safety for these patients. Our present results verify the safety of influenza vaccination in rheumatoid arthritis, confirming our own and other previous studies in SLE and RA, where patients did not experience significant clinical flares following vaccination.

The assessment of efficacy and safety of vaccination against the influenza virus in patients with rheumatoid arthritis must place special emphasis on the effect of disease-modifying anti-rheumatic drugs (DMARDs) such as tumor necrosis factor (TNF) blockers. The humoral response to influenza vaccination is not affected by the use of prednisone or DMARDs (Fomin et al. 2006).

Our study indicates that a modern influenza vaccine is well tolerated in SLE and RA patients in stable condition: in no single case was there a worsening of the main disease after immunization. Furthermore, we concluded that the patients run a much higher risk of disease exacerbation as a result of viral or bacterial infections if not vaccinated.

### Conclusions

We believe that influenza vaccine is indicated for SLE and RA patients in stable condition. However, this decision has to be made individually, on a patient-by-patient basis. We plan to continue our study with the goal of formulating a better protocol for clinical practice.

### References

Aron-Maor A, Shoenfeld Y. 2004. Vaccination and autoimmunity. In: Shoenfeld Y, Rose NR, editors. Infection and autoimmunity. New York, NY: Elsevier BV. p 105–116.

Avery RK. 1999. Vaccination of the immunosuppressed adult patient with rheumatologic disease. Rheum Dis Clin North Am 25:567–584.

Blumberg S, Bienfang D, Kantrowitz FG. 1980. A possible association between influenza vaccination and small-vessel vasculitis. Arch Intern Med 140:847–848.

Chalmers A, Scheifele D, Patterson C, Williams D, Weber J, Shuckett R, et al. 1994. Immunization of patients with rheumatoid arthritis against influenza: A study of vaccine safety and immunogenicity. J Rheumatol 21:1203–1206.

Cimmino MA, Seriolo B, Accardo S. 1995. Influenza vaccination in rheumatoid arthritis. J Rheumatol 22:1802–1803.

Gavaghan T, Webber CK. 1993. Severe systemic vasculitic syndrome post influenza vaccination. Aust NZ J Med 23:220.

Del Porto F, Laganà B, Biselli R, Donatelli I, Campitelli L, Nisini R, et al. 2006. Influenza vaccine administration in patients with systemic lupus erythematosus and rheumatoid arthritis: Safety and immunogenic. Vaccine 24(16):3217–3223.

Doran MF, Crowson CS, Pond GR, O'Fallon M, Gabriel SE. 2002. Frequency of infection in patients with rheumatoid arthritis compared with controls. A population based study. Arthritis Rheum 46:2287–2293.

Fomin I, Caspi D, Levy V, Varsano N, Shalev Y, Paran D, et al. 2006. Vaccination against influenza in rheumatoid arthritis: The effect of disease modifying drugs, including TNFα blockers. Ann Rheum Dis 65:191–194.

Glück T, Kiefmann B, Grohmann M, Falk W, Straub RH, Scholmerich J. 2005. Immune status and risk for infection in patients receiving chronic immunosuppressive therapy. Rheumatol 32:1473–1480.

Herron A, Dettleff G, Hixon B, Brandwin L, Ortbals D, Hornick R, et al. 1979. Influenza vaccination in patients with rheumatic diseases: Safety and efficacy. JAMA 242:53–56.

Iyngkaran P, Limaye V, Hill G, Henderson D, Pile KD, Rischmueller M. 2003. Rheumatoid vasculitis following influenza vaccination. Rheumatology 42:907–909.

Kurland LT, Molgaard CA, Kurland EM, Erdtmann FJ, Stebbing GET. 1984. Lack of association of swine flu vaccine and rheumatoid arthritis. Mayo Clin Proc 59:816–821.

Nosal GJV. 2000. Vaccination and autoimmunity. J Autoimmun 14:15–22.

Rose NR. 1998. The role of infection in the pathogenesis of autoimmune disease. Semin Immunol 10(1):5–13.

Shoenfeld Y, Rose NR, editors. 2004. Infection and autoimmunity. New York, NY: Elsevier BV. p 747.

Sibilia J, Maillefert JF. 2002. Vaccination and rheumatoid arthritis. Ann Rheum Dis 61:575–576.

Stojanovich L. 2005. Influenza vaccination of patients with systemic lupus erythematosus (SLE) and rheumatoid arthritis (RA) Abstract book in: VIAMR, Conference, session 2; Oct; Lausanne (CD). p 26–28.

Zandman-Goddard G, Shoenfeld Y. 2004. SLE and Infections. In: Shoenfeld Y, Rose NR, editors. Infection and autoimmunity. New York, NY: Elsevier BV. p 1491–1503.

